# Effects of individual and combined doses of added nitrite and nitrate on the quality of dry-fermented sausage

**DOI:** 10.5713/ab.24.0561

**Published:** 2025-01-24

**Authors:** Suk Nam Kang, Donggyun Yim

**Affiliations:** 1 Department of Animal Resource, Daegu University, Gyeongsan, Korea; 2 Department of Animal Science and Biotechnology, Kyungpook National University, Sangju, Korea; 3 Research Institute for Innovative Animal Science, Kyungpook National University, Sangju, Korea

**Keywords:** Color Analysis, Microbial Analysis, Residual Nitrite, Texture Analysis, Water Activity

## Abstract

**Objective:**

We aimed to investigate the impacts of nitrate and nitrite on the salami fermentation process.

**Methods:**

Experimental groups of dry-fermented sausage were prepared with 100 ppm nitrite (T1), 150 ppm nitrite (T2), and 100 ppm nitrite+50 ppm nitrate (T3). Salami quality was evaluated after a 30-day fermentation-drying process.

**Results:**

There were no significant differences in residual nitrite levels between T1 and T2, while the T3 samples exhibited the highest residual nitrite. T2 (150 ppm nitrite) showed a significantly higher pH value compared to T1 (100 ppm nitrite). Water activity was lowest in the T2 sample. T1 had a higher thiobarbituric acid reactive substances value than other treatments. In the color analysis, the L* values of T1 and T2 were significantly higher than those of T3, whereas the a* values were significantly higher in T3. In the texture analysis, T3 exhibited a higher shear force than T1 and T2. In the microbial analysis, adding 50 and 100 ppm of nitrite did not reduce the total plate count (TPC) and lactic acid bacteria count. However, T3 had a lower TPC than T1 and T2.

**Conclusion:**

The results suggest that different levels of nitrite/nitrate affect salami quality. This study is a valuable resource for understanding how using nitrite and nitrate alone or in combination affects quality in salami production.

## INTRODUCTION

Salami is a processed meat product made using salt, nitrite, and sometimes nitrate that has undergone a prolonged drying process to achieve an extended shelf life [1]. In the salting-curing process, the addition of nitrate and nitrite plays various important roles such as forming the distinctive cured meat flavor, preventing oxidative rancidity, and stabilizing the characteristic red color [2]. Salami production varies depending on multiple factors but typically takes from several weeks to several months. Adding nitrites during salami production is crucial due to their impact on meat color stability and flavor development [3]. The addition of nitrites is deemed more critical in the production of salami than in the production of heated meat products because salami is a non-heated or fresh product where harmful microbial growth can occur relatively easily, necessitating the inhibition of such growth through nitrite addition [1]. Additionally, salami contains higher concentrations of salt compared to heated meat products, which has been reported to potentially accelerate lipid oxidation in meat [4]. Nitrites effectively inhibit these oxidative reactions. For these reasons, the addition of nitrites is strongly recommended in many salami manufacturing processes. Variations in key factors such as lipid oxidation, flavor, juiciness, toughness/tenderness, and especially color can influence consumers' meat product purchasing choices [5]. So far, there is limited research regarding the impact of the addition of nitrates and nitrites and their combinations on the quality of fermented sausages. Some researchers have investigated reductions or natural alternatives to nitrate and nitrite [3,6]. However, to date, how individual nitrite and nitrate components influence outcomes remains poorly understood. Furthermore, few studies have explored the effects of non-addition or reduced addition of these substances on meat safety. There are also few publications on the combined effect of different levels of nitrates and nitrites and ripening time in salami. Therefore, this study investigated the effects of individual and combined additions of nitrate and nitrite on the physicochemical characteristics of salami.

## MATERIALS AND METHODS

### Preparation of dry-fermented sausage

Three batches of dry-fermented sausage were produced with various levels of nitrates and nitrites (Table 1). The prototype meat processing center produced low-temperature dry fermented sausages. Fresh pork loin was purchased from a local market in Geyongsan, Korea, and vacuum sealed. The back fat was thawed for 24 hours at 1°C, while the lean meat was trimmed of connective tissues and excess fat, then stored in the refrigerator. Using a 3 to 4 mm plate, the chilled pork and fat were diced and minced twice using a meat grinder (M-12S; Hankook Fujee Industries, Suwon, Korea). For fermentation, a mixed starter culture of *Lactobacillus sakei* and *Staphylococcus xylosus* (1 mL/kg) was added to the meat and thoroughly mixed with a rotary slice cutter (SF-2002; Samwoo Industry, Daegu, Korea). Each starter culture contained about 6 log colony forming units (CFU)/g, and the suspension was added to the sausage batter at 1 mL/kg. The mixture was then vacuum-packed into collagen casings (2.4 cm in diameter, 15 cm in length) using a vacuum filler (RVF 327; Düker-REX Fleischereimaschinen GmbH, Laufach, Germany). Fermentation took place in a temperature- and humidity-controlled chamber (SMK-2000SL; Metatek, Seoul, Korea) set at 23°C for the first seven days, with relative humidity between 90% to 95%. The ripening process lasted 28 days, followed by fermentation, with the temperature gradually reduced to 15°C and humidity set between 70% to 75%. Samples from each treatment were collected for physicochemical analysis on day 30 of drying and ripening. Analysis results were collected from three trials.

### Analysis of residual nitrite, pH, water activity and salinity

Residual nitrite was determined via the method described by the Association of Official Agricultural Chemists [7]. Residual nitrite assays were performed in duplicate and all treatments within a block were analyzed at the same time to minimize variation in the analysis over time. The pH value was assessed with a pH meter (MP240; Mettler Toledo, Greifensee, Switzerland). A total of 4 g salami was blended with 36 mL of distilled water and homogenized for 70 s (T25-S2; IKA, Selangor, Malaysia). The salinity of the sample was determined using the method described by Jin et al [5]. Water activity (aw) was assessed with a water activity meter (Handheld HP23-AW-A; Rotronic AG, Bassersdorf, Switzerland).

### Analysis of meat color, 2-thiobarbituric acid reactive substances and volatile basic nitrogen values

Color values were determined using a chromameter (CR-410; Minolta, Tokyo, Japan), with three replicates. Volatile basic nitrogen (VBN) contents (mg/%) were determined using the method described by Conway with slight modifications [8]. The 2-thiobarbituric acid reactive substances (TBARS) values were measured using the procedure described by Witte et al [9].

### Analysis of texture

Sample texture was assessed instrumentally using the Warner-Bratzler shear test (WBSF) and texture profile analysis (TPA) with a TA 1 Texture Analyzer (Measurement and Calibration Technologies Ametek Co., Largo, FL, USA). For the WB test, cylindrical pieces with a diameter of 1.27 cm were prepared. The pieces were cut using a WBSF shear blade with a triangular cutting edge and two parameters, work of shear and toughness, were measured. The testing procedures included a pre-test speed of 3 mm/s, a test speed of 1 mm/min, a post-test speed of 3 mm/s, and a trigger force of 10 N.

For the TPA, samples were prepared by cutting 2×2 cm cylindrical pieces and compressing them to 75% of their original height. This test utilized a 10 N load cell and a 20-mm diameter cylindrical probe. The sample was placed under the probe, which was moved downward at a pre-test speed of 3.0 mm/s, a test speed of 1.0 mm/min, and a post-test speed of 1.0 mm/s. All WBSF (work of shear and toughness) and TPA (hardness, cohesiveness, springiness, and chewiness) parameters were measured using Lloyd Instruments Ltd Nexygen/Ondio software with the Production Test program V3.0.1. Texture results were recorded in Newtons (N).

### Analysis of microbial count

Total plate counts (TPC) and lactic acid bacteria (LAB) counts were analyzed following the guidelines in the Criteria and Ingredient Standard of Livestock Products [10].

### Statistical analysis

Statistical analysis was performed using one-way analysis of variance on three replicates using SPSS (2011) program. The variance of all variables was assessed with the General Linear Model, and Duncan’s multiple range test was used to compare treatment means at a significance level of p<0.05.

## RESULTS AND DISCUSSION

### Residual nitrite of dry-fermented sausage

Table 1 shows the pH, aw, salinity, and residual nitrite content of salami after 30 days of storage with varying levels of nitrite/nitrate. Nitrate is essential during the drying process of salami, as nitrate-reducing bacteria gradually convert it into nitrite [11]. Sodium nitrate and nitrite are utilized to control different pathogenic and spoilage microorganisms, a notable example being *Clostridium botulinum* which has the potential to cause botulism, one of the most severe forms of food poisoning [12]. While sodium nitrite and nitrate are crucial for curing and preserving meat products, there is a food safety concern since sodium nitrite can lead to unwanted reactions, such as the formation of N-nitrosamines. This highlights the industrial importance of these additives and their potential toxicological effects, emphasizing the need to monitor their concentrations to minimize health risks for consumers. Thus, this study helps identify trends and factors that can influence or prevent the formation of N-nitrosamines. Practically, the formation of N-nitrosamines is minimized when sodium nitrite is added within the concentration limits set by EU legislation (Directive 2006/52/EC), which generally restricts it to 150 mg/kg of meat or meat products [13]. In this study, the T1 treatment group, which was supplemented with 100 mg/kg of nitrite during manufacture, showed the lowest nitrite content at 69.26 mg/kg. The T2 and T3 treatment groups, which were supplemented with an additional 50 ppm of nitrite and nitrate respectively, had respective nitrite contents of 71.45 mg/kg and 88.70 mg/kg. These results are consistent with the findings reported by Sallan et al [14], which indicated that the residual nitrite amount in sausages varies according to the amount of nitrite applied. Furthermore, the T3 treatment group, which was supplemented with nitrate, showed a significantly higher nitrite content compared to the nitrite-supplemented groups (p<0.05). High residual nitrite levels in combination treatments of nitrite and nitrate are significant in quality assessment because they can influence both safety and product quality. However, excessive residual nitrite poses health risks, such as the formation of carcinogenic nitrosamines. When residual nitrite levels remain high, it indicates that nitrites may not have fully reacted or degraded, which could point to an imbalance in the nitrite/nitrate ratio, improper dosing, or processing conditions that do not adequately promote their breakdown [15]. Thus, in terms of quality assessment, balancing nitrite levels is crucial to ensure both effective preservation and compliance with meat safety standards while maintaining desirable organoleptic properties.

### pH of dry-fermented sausage

During the drying and aging process of dry-fermented sausages, a decrease in pH to around 5.0 stabilizes meat color, inhibits the growth of undesirable microorganisms, and promotes the formation of desirable flavor and aroma compounds [16]. Several researchers have reported that the addition of nitrite and nitrate has various effects on texture, lowering pH and influencing product stability. Nitrite exhibits antibacterial activity associated with nitrous acid, which is most effective at low pH [17] between 4.5 and 5.5. In this study, the treatment group with increased nitrite levels, T2 (150 ppm nitrite), showed a significantly higher pH value compared to T1 (100 ppm nitrite); however, the treatment group with both nitrite and nitrate (T3) exhibited a lower pH value than T1 (p<0.01). Moreover, the treatment group using both nitrite and nitrate (T3) had slightly lower pH values compared to the treatment groups using only nitrite (T1 and T2). These results are inconsistent with previous findings that suggested nitrite and nitrate additions do not influence pH changes [18].

### Water activity and salinity of dry-fermented sausage

Aw is crucial for product safety. Values below 0.9 indicate a stable product at room temperature, preventing the growth of spoilage and pathogenic bacteria [19]. The final products of all salami samples in the present study exhibited an Aw range of 0.79 to 0.85, confirming their safety. Treatment group T2, which had increased nitrate levels, exhibited significantly lower water activity compared to T1 (p<0.05). This result indicates that the relatively low pH observed in the T3 sample was associated with a relatively high aw value. However, the T3 sample, which was treated with an additional 50 ppm nitrate, did not show a significant difference compared to the T1 treatment group. Bedale et al [20] reported in their study on the effects of nitrate and nitrite on the antioxidant activity of meat that these compounds can influence the oxidation of muscle tissue, which in turn affects water activity (Aw). Additionally, they noted that these compounds also impact microbial activity and may influence sensory characteristics as well.

Salt (sodium chloride, NaCl) is a major ingredient in dry fermented sausages, playing a critical role in ensuring microbiological stability and significantly affecting the final taste and texture. Typically, 2.0% to 4.0% NaCl is added to the sausage mince [21,22], and these concentrations increase in the final products due to the drying process. In this study, the NaCl content added during the salami manufacturing process was 2.52%. The salinity levels in the final products increased and ranged from 3.16% to 3.47% in all treatments, with no significant differences among the groups treated with different levels of nitrite/nitrate (p>0.05).

### TBARS and VBN values of dry-fermented sausage

Table 2 shows the TBARS and VBN values of the salamis after 30 days of storage with varying levels of nitrite/nitrate. The significant interaction of nitrate/nitrite dose by drying time suggests that the intensity of the oxidative phenomena over time followed a different rate of formation of malonaldehyde (MD) as affected by the level of nitrate/nitrite in the fermented sausages [23]. The lipid oxidation level was assessed by measuring TBARS developed at the end of the ripening process. Oxidation processes are a major issue because they deteriorate the quality of fermented meat products and significantly shorten their shelf life. Wójciak et al [24] reported that during a long-term storage period of 6 months, the TBARS index values ranged from 1.10 to 2.08 mg MD/kg due to the antioxidant effect of nitrate/nitrite. Specifically, lower TBARS values were observed in salamis that were supplemented with nitrate/nitrite. In this study, T1 had a relatively high TBARS value of 1.68 mg MD/kg, while the TBARS values in T2 and T3 salamis were 0.92 mg MD/kg and 0.41 mg MD/kg, respectively. These results are not in accordance with a study by Navarro et al [25] who, in dry sausages produced using a rapid fermentation process, detected a higher peroxide index in the nitrate-treated batch than in the nitrite-treated batch. Furthermore, nitrate led to a greater reduction in TBARS values compared to nitrite in salami.

Weiss et al [26] suggested that the primary functions of nitrite are to prevent the growth of foodborne pathogens and to inhibit the metabolism of proteolytic enzymes in meat products, thus decreasing their VBN values. In the present study, it was determined that the addition of 50 ppm nitrite and 50 ppm nitrate to the existing 100 ppm nitrite did not affect the inhibition of microorganisms.

### Meat color of dry-fermented sausage

Table 3 shows the meat color of the salamis after 30 days of storage with varying levels of nitrite/nitrate. Nitrite can break down into nitric oxide (NO), which reacts with myoglobin to produce pink nitrosomyoglobin during the heating of meat products [27]. In this study, there were no differences in lightness (L* value) and redness (a* value) between T1 and T2. Higuero et al [18] showed that 75 ppm nitrate or 37.5 ppm nitrite produced a similar color to that seen with 150 ppm nitrate or 150 ppm nitrite treatment. However, in the present study, T3, which was supplemented with nitrate, had lower lightness (p<0.01) but higher redness (p<0.05) compared to T1 and T2. This result is consistent with the reports of many researchers indicating that nitrite and nitrate affect the red color of cured meat products [28]. In our study, nitrate had a greater impact on redness compared to nitrite. However, there were no significant differences in yellowness (b* value) between the samples.

### Texture analysis of dry-fermented sausage

Table 4 shows the WBSF and TPA of salami after 30 days of storage with varying levels of nitrite/nitrate. There were no significant differences in WBSF between T1 and T2; however, T3 exhibited a significantly higher WBSF compared to the other treatments. These results indicate that the addition of nitrate to the baseline sample (with 100 ppm nitrite) led to increased WBSF in texture compared to the addition of nitrite, demonstrating its impact on reducing the tenderness of salami. However, in the TPA, no significant differences were observed in hardness, cohesiveness, springiness, or adhesiveness among the treatments. Zhang et al [28] emphasized that nitrite has a significant impact on the interactions between meat proteins, particularly the muscle proteins myosin and actin, in fermented meat products. They reported that nitrite influences protein denaturation and aggregation, which enhances the texture and cohesiveness of fermented meats. This, in turn, also affects both the sensory and structural properties of the products.

### Microbial analysis of dry-fermented sausage

Table 5 shows the TPC and LAB of salami after 30 days of storage with varying levels of nitrites/nitrates. Marco et al [29] reported that nitrite inhibits spoilage and pathogenic bacteria, such as *Clostridium botulinum*, while selectively promoting LAB. This selective pressure enhances the safety and quality of fermented sausages by allowing LAB to thrive and suppress undesirable microorganisms. Interestingly, microbial analysis demonstrated that T3 had a significantly lower TPC value (9.62 log CFU/g) compared to the other treatments (T1 and T2) (p<0.05). These results are similar to the findings of Dalzini et al [30], who reported that the combined use of nitrites and nitrates in salami results in a lower total bacterial count compared to the use of nitrites alone. This is attributed to the synergistic effect of these compounds in inhibiting pathogenic and spoilage microorganisms. However, there were no significant differences in LAB among the treatments in the present study. These results suggest that the addition of nitrite does not affect TPC or LAB, whereas the addition of nitrate leads to a reduction in TPC but does not influence LAB. These results differ from previous studies that reported an increase in nitrate and nitrite led to a reduction in lactic acid bacteria counts [15].

## CONCLUSION

In conclusion, there were no significant differences in residual nitrite content between the 100 ppm (T1) and 150 ppm (T2) nitrite additions, while the addition of 100 ppm nitrite resulted in the lowest aw value. The L* values were higher in the nitrite-added treatment groups (T1 and T2) compared to the group treated with nitrate (T3), whereas the a* values were higher in the 100 ppm nitrite+50 ppm nitrate treatment (T3) group compared to the 100 ppm nitrite-only treatment (T1) group. Additionally, T3 exhibited the highest shear force value and lowest TPC values among samples. Furthermore, this study indicated that the addition of 50 and 100 ppm of nitrite does not affect the reduction of TPC and LAB. Through these findings, we provide basic data that will enable meat processors to purposefully combine sodium nitrate and nitrite in cured meat and meat products. This research is expected to assist in maintaining more stringent regulations.

## Figures and Tables

**Table 1:** Salami formulations including nitrite and nitrate

Ingredient (%)	Treatments^1)^		

	T1	T2	T3
Pork lean meat	74.77	74.77	74.77
Pork backfat	13.08	13.08	13.08
Water (ice)	6.98	6.98	6.98
Nitrite	0.01	0.015	0.01
Nitrate	0.00	0.00	0.005
Salt	2.52	2.52	2.52
Sodium ascorbate	0.19	0.19	0.19
Sugar	0.56	0.56	0.56
Glucose	0.56	0.56	0.56
Black pepper	0.19	0.19	0.19
Garlic powder	0.37	0.37	0.37
Seasoning	0.49	0.49	0.49
Starter+milk	0.30	0.30	0.30
Total	100	100	100

1) T1, 100 ppm nitrite; T2, 150 ppm nitrite; T3, 100 ppm nitrite+50 ppm nitrate.

**Table 2:** Physicochemical parameters of salamis prepared with different concentrations of nitrate and nitrite

	T1^1)^	T2	T3	p-value	SEM
Nitrite (mg/kg)	69.26±1.10^b^	71.45±4.99^b^	88.70±8.27^a^	0.01	2.80
pH	4.26±0.01^b^	4.32±0.01^a^	4.22±0.00^c^	0.01	0.00
Aw	0.85±0.01^a^	0.79±0.00^b^	0.83±0.02^a^	0.01	0.01
Salinity (%)	3.16±0.07	3.47±0.04	3.44±0.06		0.79
TBARS (mg MD/kg)	1.68±0.01^a^	0.92±0.03^b^	0.41±0.01^c^	0.01	0.01
VBN (mg%)	42.62±1.62	46.22±2.85	45.42±5.14		2.04

1) T1, 100 ppm nitrite; T2, 150 ppm nitrite; T3, 100 ppm nitrite+50 ppm nitrate.

a–c Superscripts with different letters within the same row differ significantly (p<0.05).

SEM, standard error of the mean; Aw, water activity; TBARS, thiobarbituric acid reactive substances; MD, malonaldehyde; VBN, volatile basic nitrogen.

**Table 3:** Color parameters of salamis prepared with different concentrations of nitrate and nitrite

	T1^1)^	T2	T3	p-value	SEM
L*	54.80±0.94^a^	55.84±1.13^a^	47.55±1.39^b^	0.01	0.51
a*	12.13±0.52^b^	13.23±0.70^ab^	14.07±1.29^a^	0.05	0.47
b*	8.54±0.69	8.54±0.68	8.68±1.12		0.34

1) T1, 100 ppm nitrite; T2, 150 ppm nitrite; T3, 100 ppm nitrite+50 ppm nitrate.

a,b Superscripts with different letters within the same row differ significantly (p<0.05).

SEM, standard error of the mean.

**Table 4:** Texture parameters of salamis prepared with different concentrations of nitrate and nitrite

	T1	T2	T3	p-value	SEM
WBSF (N)	2.74±0.17^b^	2.22±0.20^b^	4.75±0.39^a^	0.01	0.18
Hardness (N)	13,848.08±1,724.48	19,918.00±6,072.73	11,718.80±2,672.41		2485.07
Cohesiveness	0.95±0.13	0.94±0.04	0.92±0.04		0.02
Springiness	0.35±0.06	0.33±0.01	0.33±0.03		0.17
Chewiness (kgf)	4419.20±149.60	6107.66±1722.46	3501.24±522.58		689.71
Adhesiveness	8.29±1.53	8.02±2.88	6.05±2.99		1.88

1) T1, 100 ppm nitrite; T2, 150 ppm nitrite; T3, 100 ppm nitrite+50 ppm nitrate.

a,b Superscripts with different letters within the same row differ significantly (p<0.05).

SEM, standard error of the mean; WBSF, Warner-Bratzler shear test.

**Table 5:** Microbial counts in salamis prepared with different concentrations of nitrate and nitrite

	T1	T2	T3	p-value	SEM
TPC (log CFU/g)	10.04±0.06^a^	9.96±0.10^a^	9.62±0.20^b^	0.05	0.10
LAB (log CFU/g)	10.21±0.16	10.13±0.11	9.89±0.08		0.09

1) T1, 100 ppm nitrite; T2, 150 ppm nitrite; T3, 100 ppm nitrite+50 ppm nitrate.

a,b Superscripts with different letters within the same row differ significantly (p<0.05).

SEM, standard error of the mean; TPC, total plate count; CFU, colony forming units; LAB, lactic acid bacteria.
